# Ratio of plasma IL-13/TNF- ∝ and CXCL10/CCL17 predicts mepolizumab and omalizumab response in asthma better than eosinophil count or immunoglobulin E level

**DOI:** 10.1038/s41598-024-60864-3

**Published:** 2024-05-06

**Authors:** Ayobami Akenroye, Tanawin Nopsopon, Jonathan J. Hacker, Tanya M. Laidlaw

**Affiliations:** 1 Division of Allergy and Clinical Immunology, Department of Medicine, Brigham and Women’s Hospital and Harvard Medical School, 60 Fenwood Road, Boston, MA 02115 USA; 2 Channing Division of Network Medicine, Department of Medicine, Brigham and Women’s Hospital and Harvard Medical School, Boston, MA USA

**Keywords:** Predictive markers, Outcomes research, Biological therapy

## Abstract

To date, most studies to identify biomarkers associated with response to the anti-interleukin 5 agent, mepolizumab, and to the anti-immunoglobulin E agent, omalizumab have focused on clinically available biomarkers, such as the peripheral blood eosinophil counts (BEC) and total immunoglobulin E (IgE). However, these biomarkers often have low predictive accuracy, with many patients with eosinophilic or allergic asthma failing to demonstrate clinical response to mepolizumab or omalizumab respectively. In this study, we evaluated the association of baseline pre-biologic plasma levels of 26 cytokines and chemokines, including T-helper 1 (Th1)-, Th2-, Th17-related cytokines, and their ratios with subsequent clinical response to mepolizumab or omalizumab. We defined clinical response as a reduction in the baseline annual exacerbation rate by half or more over the one-year period following initiation of the biologic. Baseline levels of plasma IL-13 were differentially elevated in responders versus non-responders to mepolizumab and plasma CXCL10 levels were differentially elevated in responders to omalizumab. The ratio of IL-13/TNF-α had the best sensitivity and specificity in predicting response to mepolizumab and CXCL10/CCL17 to omalizumab, and these performed better as predictive biomarkers of response than BEC and IgE. Cytokines and chemokines associated with airway eosinophilia, allergic inflammation, or Th2 inflammation, such as IL-13 and CXCL10, may be better predictors of clinical response to mepolizumab and omalizumab, than IL-5 or IgE, the targets of mepolizumab and omalizumab.

## Introduction

Over the past two decades, six monoclonal antibodies “biologics” have been approved for the treatment of severe asthma uncontrolled on conventional therapy^1^. In randomized trials and in clinical cohorts, these biologics significantly reduced asthma exacerbations, improved lung function, and improved overall asthma-related quality of life^2,3^. The potential value of these biologics is however not being maximized given the paucity of accurate predictors to inform therapeutic decisions on which biologic a patient is likely, or unlikely, to respond to. This challenge has become more important as more biologics are rapidly introduced to the market- five of these six biologics were approved in the six-year period between 2015 and 2021- and given the high percentage of patients eligible for two or more of these therapies^4^. The current approach includes selection of an initial biologic based on provider choice and switching from one biologic to the another if a patient demonstrates suboptimal response^5,6^.This trial-and-error approach is frustrating to patients, challenging to providers, and can lead to potentially fatal delays in optimizing asthma control in this high-risk patients. Furthermore, this approach is costly given that these biologics are expensive and cost about $30,000-$40,000 per patient annually^7^. Thus, the identification of biomarkers to differentiate patients likely to respond to each of these therapies from those unlikely to respond is potentially of great clinical value to patients, their providers, and to the health system.

Omalizumab, an anti-immunoglobulin E antibody, and mepolizumab, which blocks interleukin 5 (IL-5), a major player in eosinophilic inflammation, were the first FDA-approved respiratory biologics and two of the most used respiratory biologics worldwide. Although a few clinical biomarkers have been shown to be associated with omalizumab and/or mepolizumab response, these biomarkers have limited predictive power and are also likely to be associated with response to the other biologics. For instance, mepolizumab’s efficacy increases as peripheral eosinophil count increases^8^. However, not all asthmatic patients with peripheral eosinophilia demonstrate clinical response to mepolizumab^8^. One possible explanation for this is the lack of congruence between peripheral and airway eosinophilia. Thus, a biomarker that correlates better with airway tissue eosinophilia may be a better predictor of mepolizumab’s response. Additionally, given that the immune system is complex and abounds with interactions between cytokines and chemokines, the most helpful biomarkers may be those involving balance between two or more biomarkers.

Asthma has historically been considered a ‘type 2’ inflammatory disease involving cytokines produced by T-helper (Th)-2 lymphocytes and innate lymphoid cell type 2 (ILC2). However, there is growing evidence that many patients with asthma demonstrate a mixed endotype of type 1 and type 2 inflammation, and that the balance between Th2- and Th1- or Th17-related cytokines is associated with asthma development, phenotype, disease severity, asthma exacerbations, and response to corticosteroid treatment.^9–18^ In this study, we evaluated the association between Th1-, Th2-, and Th17-related cytokines and chemokines and their ratios with subsequent clinical response to omalizumab and mepolizumab.

## Methods

### Study population and data extraction

We identified the first 39 monoclonal antibody-naïve patients with severe asthma who initiated omalizumab (n = 21) or mepolizumab (n = 18) between April 2015 and July 2022, had plasma samples in the Mass General Brigham (MGB) Biobank collected prior to biologic therapy initiation but not during an acute exacerbation, and who had no other indication for omalizumab or mepolizumab^1^. The MGB Biobank collects and stores blood samples from patients within the MGB health system in Boston, MA and provides these samples to researchers within the MGB system for a nominal fee following approval by the MGB Institutional Review Board (IRB)^19^. We defined clinical response as a reduction in the baseline annual exacerbation rate by at least half over the one-year period following initiation of omalizumab or mepolizumab^20^. Exacerbations were defined as an emergency room visit or hospitalization with a primary diagnosis of asthma or an ambulatory visit with a prescription of oral corticosteroids for ≥ 3 days. Steroid prescriptions within 7 days of each other were counted as a single exacerbation episode^3,21^. Patients were followed from therapy initiation to 12 months following initiation or to the end of administrative follow-up (December 2022). Individuals who switched to another biologic were censored at the point of switching to avoid misappropriately attributing their outcome to the index biologic. Clinical characteristics of the cohort were extracted electronically from the medical records with additional manual chart reviews conducted when data were missing. This was a non-experimental study approved by the MGB IRB and conformed to the guiding principles of Human Subjects Research^22^.

### Proteomics assay of cytokines and chemokines

We conducted a high-throughput aptamer-based proteomics assay to evaluate 26 cytokines and chemokines selected from a list of 6,600 possible human protein targets using the SomaScan assay^23,24^. This included alarmins which stimulate the release of Th2-related cytokines (thymic stromal lymphopoietin (TSLP) and IL-25/IL-17E), canonical Th2 cytokines (IL-4, 5, 9, 13), Th1-type cytokines (interferon gamma (IFN-), tumor necrosis alpha (TNF-α), Th1-attracting (IFN-gamma-inducible protein 10 (IP-10)/CXCL10 ), Th2-attracting chemokines (thymus and activation-regulated chemokine (TARC)/CCL17), macrophage-derived chemokine (MDC)/CCL22), I-309/CCL1), Th17-associated cytokines or cytokines which stimulate neutrophils to produce IL-17 (IL-17A-F, IL21, IL-22, IL-23), or Th-17 chemoattractant (Gro-alpha/CXCL-1 and IL-8/CXCL-8), and other pro-inflammatory cytokines including IL-6, IL-21, IL-1α, IL-1ß, and the immunomodulatory cytokine, IL-10^25–30^.

The technique of the SomaScan assay by Somalogic and the quality control processes have been previously described^23,24^. Briefly, modified aptamers (SOMAmer reagents) bind to the protein targets in the plasma sample. Non-specifically bound SOMAmer reagents are removed in multiple washing steps and readout in relative fluorescent units (RFU) are done using Agilent hybridization, scan, and feature extraction technology. Experimental controls are added to each 96-well plate for quality control and calibration, controlling for batch effects and to estimate the accuracy and the background buffer effects of the assay. Twelve Hybridization Control SOMAmer reagents are added alongside the SOMAmer reagents and are measured from both the biological samples and the experimental controls to control for readout variability. Data standardization with signal normalization to sample-specific signals within dilution bins and calibration are done. Total coefficients of variation (CV) are calculated and expected to be less than 20% for all analytes demonstrating that technical bias is mitigated. In general, SomaScan’s CV was about ~ 5% for all targets of interest.

### Descriptive analyses

We describe the clinical characteristics of the cohort at baseline using means and standard deviations or medians and interquartile ranges for continuous variables and proportions for categorical variables. In exploratory analyses, we also evaluated clinical characteristics by response status. Thereafter, we evaluated statistical correlation between each pair of proteins at baseline using Spearman correlation in responders versus nonresponders to identify protein relationships that are different by response status. Correlation coefficients with absolute values of 0.8 or above (≤ − 0.8 or ≥  + 0.80) were considered to be ‘very strong’, 0.6–0.7 as ‘moderate’, 0.3–0.5 as ‘fair’, and 0.1–0.2 as ‘poor’^31^.

### Differential expression analyses

We followed standard protocols for analyzing proteomics data^23,24^. Prior to analyses, protein expression levels were natural log transformed and median signal normalized to a reference by adaptive normalization with maximum likelihood within the dilution group. The expression levels were reported in Relative Fluorescence Units. Using the limma package in R^32^, we conducted differential expression analyses comparing the pre-treatment plasma levels of each of the 26 proteins at omalizumab or mepolizumab initiation to identify top cytokines with highest differential expression between responders and nonresponders. All models were adjusted for the baseline pre-treatment exacerbation rate. Then, we evaluated the association of protein ratios with response using limma. Given our low sample size, we did not adjust for multiple testing. In sensitivity analyses, we adjusted for the lag between sample collection and initiation of therapy.

### Exploratory analyses benchmarking the predictive accuracy of cytokines and cytokine ratios against the baseline eosinophil count or IgE level

Given that the peripheral eosinophil count is associated with mepolizumab response and that IgE levels may be associated with response to omalizumab^33,34^, we sought to evaluate if plasma cytokines or chemokines in the top ratios associated with response would have better predictive accuracy than the baseline eosinophil count in mepolizumab users or than the pre-treatment IgE level in omalizumab users. We used pre-defined bounds of 150 and 300 cells per microliter for eosinophil count, thresholds that have used to define eosinophilic asthma and response to mepolizumab, and a cutoff of 100 ku/L for total IgE^33^. For each evaluated biomarker, that is cytokine or chemokine levels or their ratios, we calculated the specificity, sensitivity, and accuracy. We constructed the receiver operating characteristic (ROC) curve using the range of sensitivity and specificity from all possible cut-points to categorize patients as responders or non-responders. Thereafter, we identified the optimal cut-point that optimized the sensitivity and specificity of each biomarker and highlighted the biomarker(s) with the highest area under the curve (AUC). If there was more than one optimal cut-point, the median value of the optimal cut-off points was selected. Lastly, we benchmarked the performance of the biomarkers in the mepolizumab group against the baseline eosinophil count ≥ 150 or 300 cells/μL and against maximum eosinophil count within three years ≥ 300 cells/μL. For the omalizumab group, we benchmarked the performance of these biomarkers against the performance of the baseline IgE level ≥ 100 IU/mL.

## Results

### Characteristics of the study population

The average age of patients in the mepolizumab group was 55.9 years, 72% were females, patients were obese on average and had about 3.0 exacerbations in the prior year. The median peripheral eosinophil count was 244 cells per microliter at baseline [interquartile range, IQR, 69–362] (Table 1). The average age of patients in the omalizumab group was 45.2 years, 86% were females, patients were overweight on average and had on average 2.1 exacerbations in the prior year. The median IgE level was 87 cells per microliter [IQR, 38–266] (Table 1.) Seven (39%) of the 18 mepolizumab patients and 7 (33%) of the 21 omalizumab patients met criteria for clinical response. All patients were still on biologic therapy at the time of the clinical outcome assessment. The follow-up period was a median of 365 days in both the omalizumab group (IQR: 365–365 days; range 173–365 days) and in the mepolizumab group (IQR: 365–365 days; range 135–365 days). No omalizumab patient switched during therapy. Two mepolizumab patients switched to another biologic on day 135 and day 355. The median time to sample collection was 1 year for all patients with 30% of patients having samples within 6 months of therapy initiation. Non-responders to mepolizumab were mostly females and both mepolizumab and omalizumab responders had higher baseline IgE levels (Supplemental Table S1).

**Table 1:** Baseline characteristics of participants.

	Mepolizumab	Omalizumab
N	18	21
Age in years, mean (SD)	55.9 (12.0)	45.2 (15.2)
Female, n (%)	13 (72)	18 (86)
White race, n (%)	13 (72)	19 (91)
Current smoker, n (%)	1 (5.6)	0 (0.0)
Former smoker, n (%)	5 (27.8)	3 (14.3)
BMI, kg/m^2^; mean (SD)	30.5 (7.3)	29.4 (8.0)
Baseline annualized exacerbation rate, mean (SD)	3.0 (1.9)	2.1 (1.6)
Baseline pre-bronchodilator FEV1, L; mean (SD)	2.0 (0.85)	2.5 (0.85)
Baseline pre-bronchodilator FEV1% predicted, mean (SD)	70 (23)	86 (20)
Peripheral blood eosinophil counts, cells/µL; median [IQR]^a^		
*At biologic initiation*	244 [69–362]	126 [72–280]
*Maximum count in the 3 years before therapy initiation*	600 [381–1210]	220 [90–335]
Immunoglobulin E (IgE), IU/µL; median [IQR]^a^	215 [50–482]	87 [38–266]
Allergic rhinitis, n (%)	15 (83)	21 (100)
Atopic dermatitis, n (5)	0 (0)	0 (0)
COPD, n (%)	1 (6)	0 (0)

BMI, Body mass index; COPD, chronic obstructive pulmonary disease; FEV1, forced expiratory volume in one second; IQR, interquartile range; SD, standard deviation.

^a^One omalizumab user was missing baseline blood eosinophil counts and one mepolizumab user with missing baseline IgE.

### Differential expression analyses

**Figure 1:**
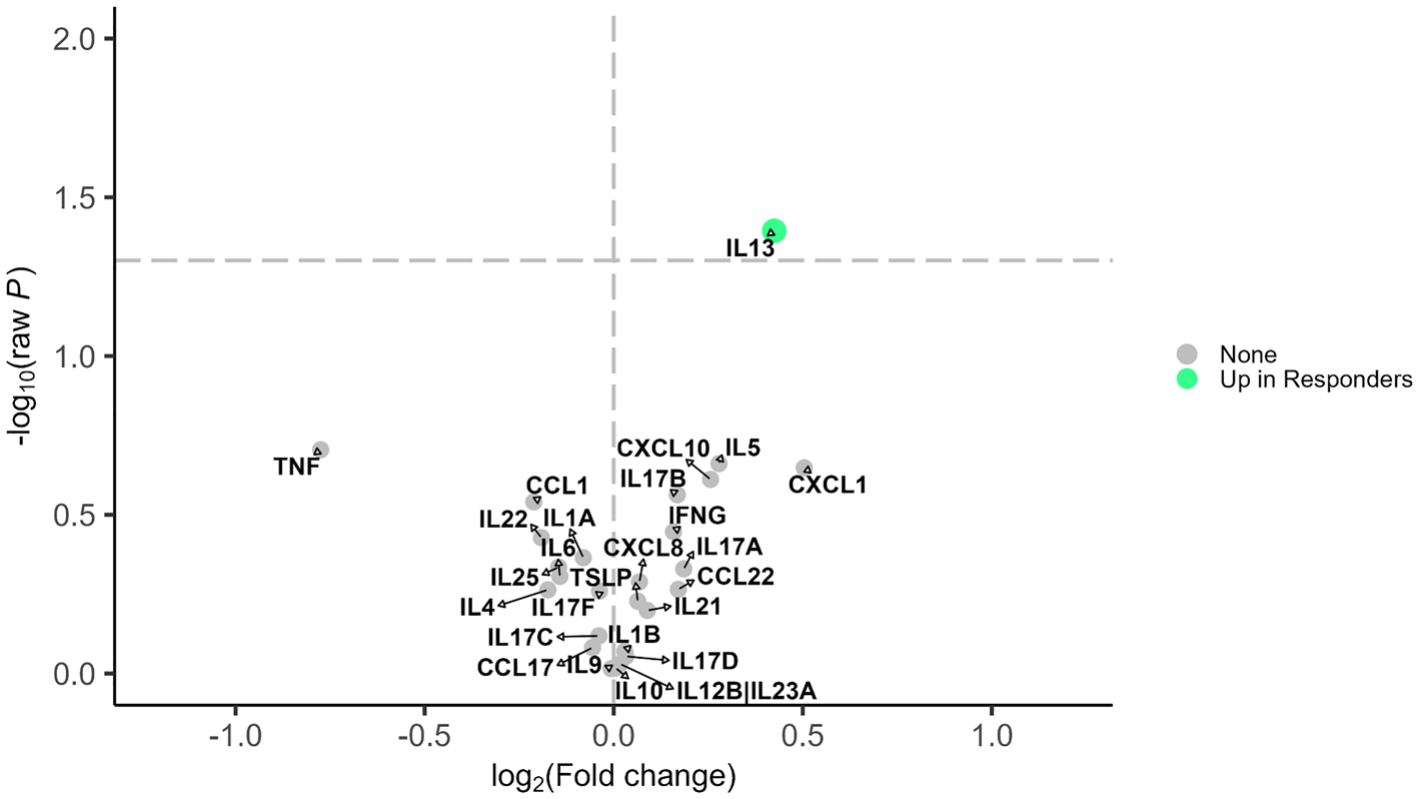
Differential expression analysis for 26 cytokines and response status in the mepolizumab group. Shows the results from limma analysis adjusted for pre-treatment annualized exacerbation rate. Cytokines with raw *P*-value < 0.05 were considered statistically significant. Effect sizes were presented as log2 of fold change where positive (negative) values indicated up-regulation in responders (non-responders). The vertical dashed line represents no difference. The horizontal dashed line represents *P*-value < 0.05. *Green dot indicates levels of the cytokine are higher in responders; *Gray dot ‘None’ indicates levels of that cytokine are not different between responders and nonresponders*.*

**Figure 2:**
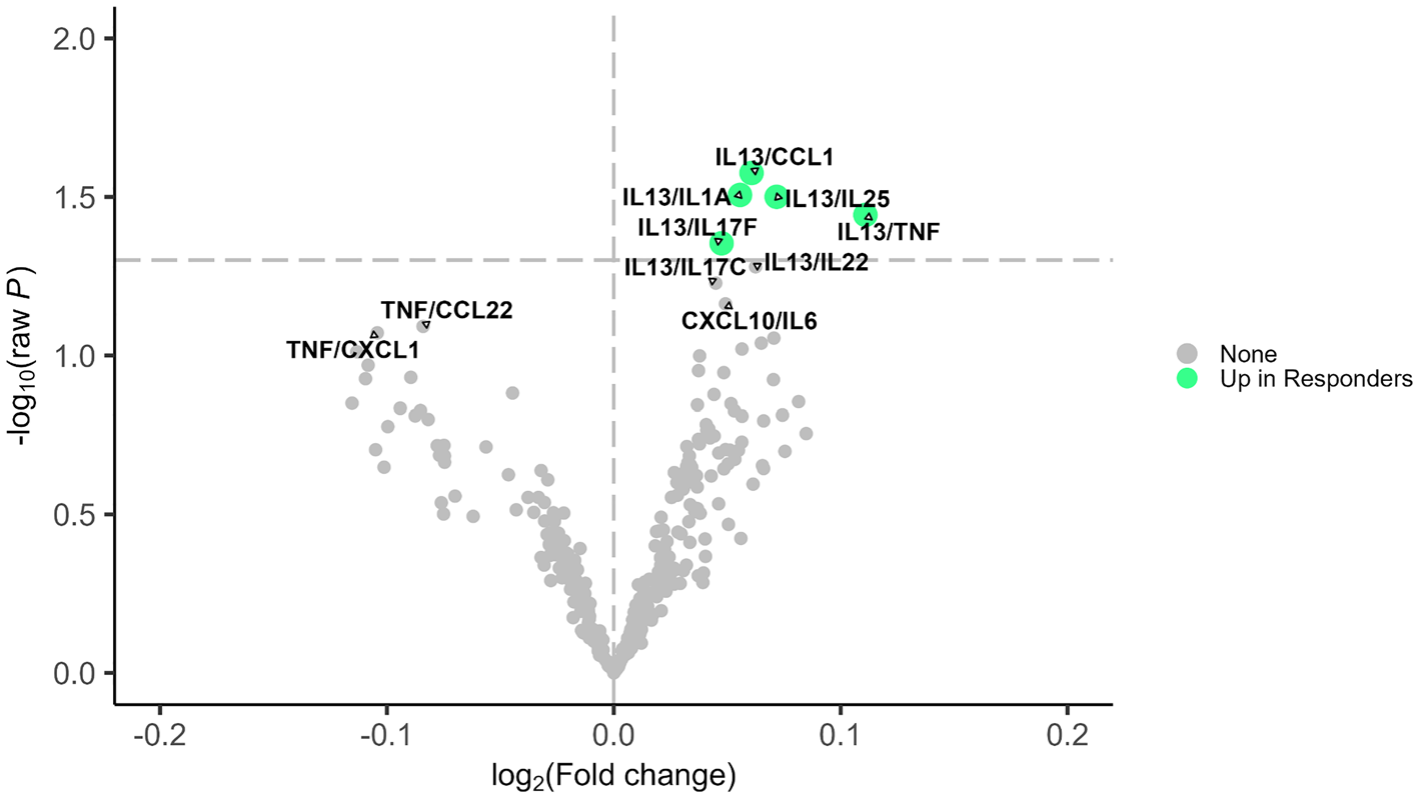
Differential expression analysis for ratios of cytokines/chemokines and response status in the mepolizumab group. Shows the results from limma analysis adjusted for pre-treatment annualized exacerbation rate. Cytokine ratios with raw *P*-value < 0.05 were considered statistically significant. Effect sizes are presented as log2 of fold change where positive (negative) values indicate up-regulation in responders (non-responders). The vertical dashed line represents no difference. The horizontal dashed line represents *P*-value < 0.05. Green dot indicates levels of the cytokine are higher in responders; Gray dot “None” indicates levels of that cytokine are not different between responders and nonresponders*.*

For mepolizumab, IL-13 was significantly higher in responders (Fig. 1 and Table S2). Levels of IL-5 were also higher in responders at baseline compared to non-responders and levels of TNF-α were lower in responders though these differences did not reach statistical significance. In differential expression analyses comparing ratios of cytokine pairs, adjusting for baseline exacerbation, the ratios of IL-13 to TNF-α, CCL1, IL-1α, IL-25, and to IL-17F were all significantly higher in responders (Fig. 2 and Table S3).

**Figure 3:**
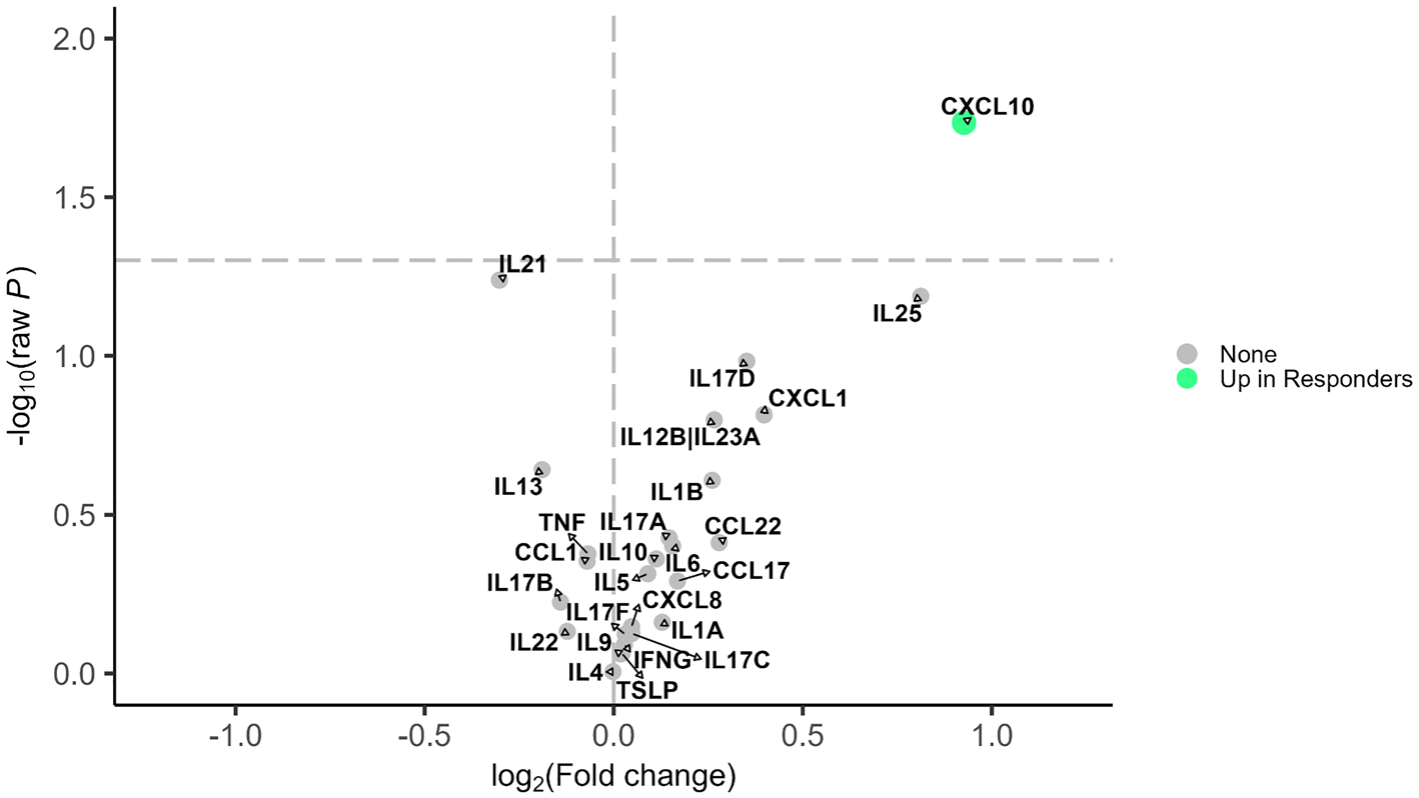
Differential expression analysis for 26 cytokines and response status in the omalizumab group. Shows the differential expression between omalizumab responders and non-responders from limma analysis adjusted for pre-treatment annualized exacerbation rate. Cytokines with raw *P*-value < 0.05 were considered statistically significant. Effect sizes are presented as log2 of fold change where positive (negative) values indicate up-regulation in responders (non-responders). The vertical dashed line represents no difference. The horizontal dashed line represents *P*-value < 0.05. Green dot indicates levels of the cytokine are higher in responders; Gray dot “None” indicates levels of that cytokine are not different between responders and nonresponders*.*

**Figure 4:**
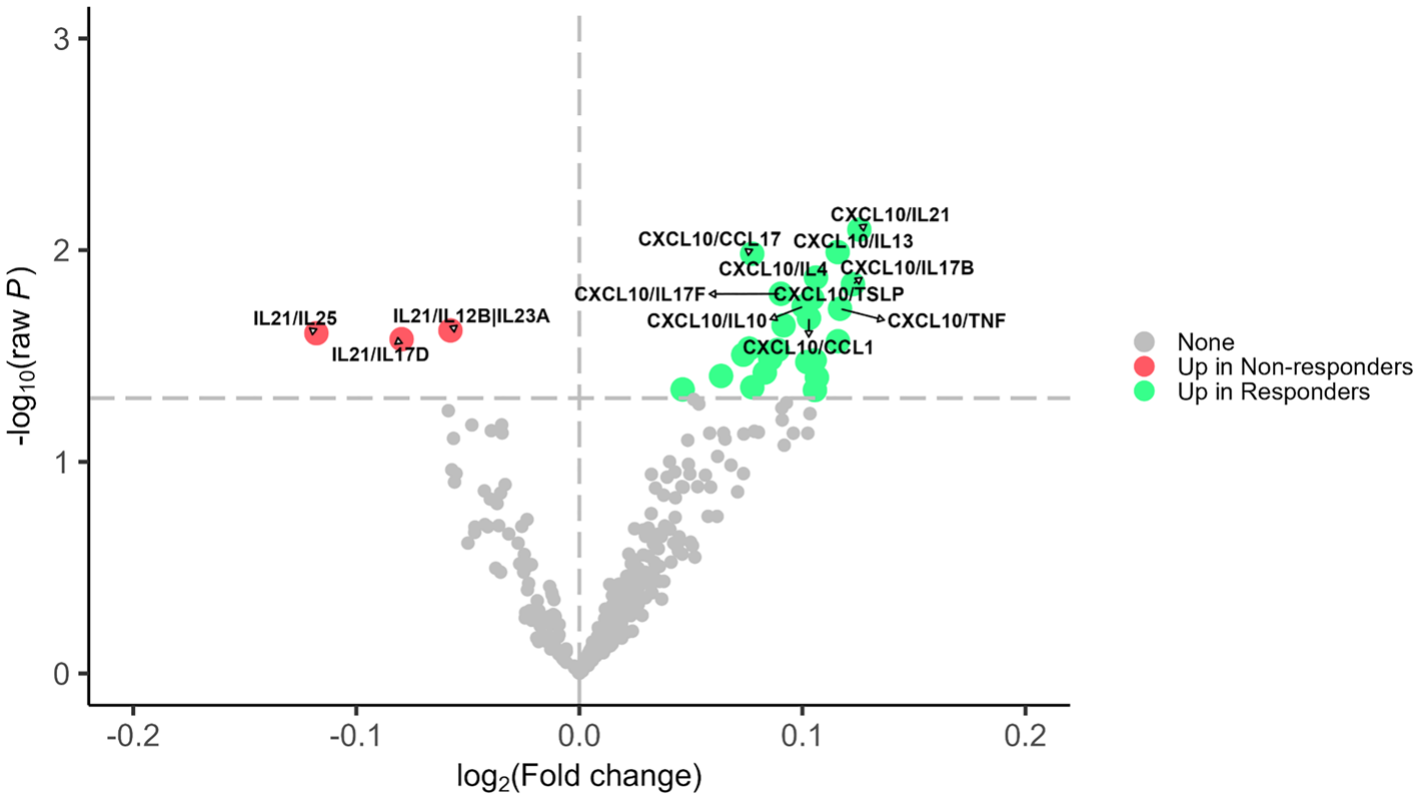
Differential expression analysis for ratios of cytokines/chemokines and response status in the omalizumab group. Shows the results from limma analysis adjusted for pre-treatment annualized exacerbation rate. Cytokine ratios with raw *P*-value < 0.05 were considered statistically significant. Effect sizes were presented as log2 of fold change where positive (negative) values indicated up-regulation responders (non-responders). The vertical dashed lines represented no difference. The horizontal dashed line represented *P*-value < 0.05. Green dot indicates levels of the cytokine are higher in responders; Red circle with triangle indicates levels of the cytokine are higher in nonresponders (or lower in responders); Gray dot “None” indicates levels of that cytokine are not different between responders and nonresponders.

For omalizumab, higher levels of CXCL10 were significantly higher in responders (Fig. 3 and Table S4). Levels of IL-21 was lower in responders, but this difference did not reach statistical significance (*p* = 0.06). In differential expression analyses comparing ratios of cytokine pairs, adjusting for baseline exacerbation, the ratios of IP-10 to multiple other cytokines and chemokines were significantly different between responders and nonresponders with CXCL10 to CCL17, CXCL10 to IL-21, and CXCL10 to IL-13 the top ratios which were significantly higher in responders and IL-21 to IL-25, IL-21 to IL-23, and IL-21 to IL-17D significantly lower in responders (Fig. 4 and Table S5).

Results adjusting for the lag between sample collection and therapy initiation were consistent with the main analyses showing that IL-13 was the cytokine most predictive of mepolizumab response and CXCL-10 of omalizumab response (Supplemental Figure S1 and S2).

### Correlations between cytokines and chemokines at baseline by response status

For responders to mepolizumab, at baseline, multiple cytokine or chemokine pairs demonstrated strong negative correlations. These included IL-17C and IL-10 (Spearman correlation coefficient, ρ: − 0.96), TSLP and IL-23 (ρ: − 0.86), IL-25 and IL-23 (ρ: − 0.86), IL-17A and CCL22 (ρ: − 0.82), CCL17 and IL-1β (ρ: − 0.82), and IL-13 and CXCL10 levels (ρ: − 0.82), (Supplemental Figure S3). In non-responders, these negative correlations were either ‘fair’ to ‘poor’ (IL-13 and CXCL10; IL-25 and IL-23; CCL17 and IL-1β) or reversed with positive correlations (IL-17C and IL-10, TSLP and IL-23, IL-17A and CCL22). TSLP levels were strongly positively correlated with IL-17A and IL-6 levels in responders but only moderately or poorly correlated in nonresponders. Contrariwise, IL-4 and TNF-α were strongly negatively correlated in nonresponders but only poorly correlated in responders (Supplemental Figure S4).

In omalizumab responders, CCL1 had a perfect negative correlation with CXCL1 (ρ: − 1.00) and very strong negative correlations with CCL17 (ρ: − 0.96) (Supplemental Figure S4). However, in nonresponders there was a positive correlation between CCL1 and CXCL1 and poor negative correlation between CCL1 and CCL17. Similarly, the strong negative correlation between both TNF-α and CXCL1 and between IFN-g and CXCL1 were poor positive correlations in nonresponders. All positive correlations in responders were also positive correlations in nonresponders. However, only 2 of the 11 strong positive correlations between cytokines or chemokines in responders demonstrated a similar degree of strength in nonresponders. Similarly, IL-17F and IL-6 were strongly negatively correlated in nonresponders (ρ: − 0.80) but were positively correlated in responders (Supplemental Figure S4).

### Exploratory analyses benchmarking the predictive accuracy of cytokines and cytokine ratios against the baseline eosinophil count or IgE level

For mepolizumab, we evaluated the predictive accuracy of the ratios of IL-13 to CCL1, IL-1α, IL-25, TNF-α, and IL-17F, which were all significantly associated with response in differential expression analyses, to the predictive accuracy of the peripheral eosinophil count at baseline. Using an eosinophil cut-off of ≥ 150 and ≥ 300 cells/μl at baseline to identify potential responders to mepolizumab had a sensitivity of 86% and 71% respectively. However, specificity using the cut-off of eosinophils ≥ 150 cells/μl was 55% while that using the cut-off of eosinophils ≥ 300 cells/μl was 82% (Table 2 and Supplemental Figure S5). Combining IL-13 levels or the ratio of IL-13 to IL-13 to TNF-α, IL-25, or IL-17F, improved specificity of using ≥ 300 cells/μl but the sensitivity remained unchanged (Table 2). Likewise, when IL-13/IL-25 or IL-13/IL-17F was used in conjunction with eosinophil ≥ 150 to define response, the specificity improved significantly (from 55 to 91%) but the sensitivity decreased from 86 to 71%. The single biomarker that maximized sensitivity and specificity was IL13/TNF- ∝ that had sensitivity and specificity both above ≥ 82% (Table 2 and Supplemental Figure S5).

**Table 2:** Sensitivity, specificity, and accuracy of predicting mepolizumab response using cutoffs defined by the top cytokine or ratios.

*Measures used in cutoff	Sensitivity	Specificity	Accuracy	AUC
Baseline Eos ≥ 300 + IL-13	0.71	1.00	0.89	0.83
Baseline Eos ≥ 300 + IL-13/IL-25	0.71	1.00	0.89	0.83
Baseline Eos ≥ 300 + IL-13/TNF- ∝	0.71	1.00	0.89	0.83
Baseline Eos ≥ 300 + IL-13/IL-17F	0.71	1.00	0.89	0.83
IL13/TNF- ∝	0.86	0.82	0.83	0.88
Max Eos ≥ 300 + IL-13/TNF- ∝	0.86	0.82	0.83	0.90
Max Eos ≥ 300 + IL-13/IL-1 ∝	0.71	0.91	0.83	0.88
Baseline Eos ≥ 150 + IL-13/IL-25	0.71	0.91	0.83	0.84
Baseline Eos ≥ 150 + IL-13/IL-17F	0.71	0.91	0.83	0.84
IL13/IL1 ∝	0.71	0.91	0.83	0.84
Baseline Eos ≥ 300 + IL-13/IL-1 ∝	0.57	1.00	0.83	0.83
Max Eos ≥ 300 + IL-13	0.86	0.73	0.78	0.84
Baseline Eos ≥ 300	0.71	0.82	0.78	0.77
Max Eos ≥ 300 + IL-13/IL-25	0.71	0.82	0.78	0.88
Baseline Eos ≥ 150 + IL-13/TNF- ∝	0.71	0.82	0.78	0.82
Max Eos ≥ 300 + IL-13/IL-17F	0.71	0.82	0.78	0.88
IL-13/IL-25	0.71	0.82	0.78	0.82
IL-13/IL-17F	0.71	0.82	0.78	0.86
Baseline Eos ≥ 150 + IL-13/IL-1 ∝	0.57	0.91	0.78	0.82
IL-13	0.86	0.64	0.72	0.79
Baseline Eos ≥ 150 + IL-13	0.71	0.73	0.72	0.78
Baseline Eos ≥ 150	0.86	0.55	0.67	0.70
Max Eos ≥ 300	1.00	0.36	0.61	0.68

*Eos: eosinophil count in cells/microliter; Max Eos: maximum eosinophil count in the 3 years prior; Cytokine or chemokine cut-off points are at the point that maximizes predictive accuracy.

For omalizumab, the sensitivity of all measures including the baseline IgE, CXCL10, and CXCL10 to IL-4, IL-13, IL-17B, IL-21 ratios, which were significantly associated with response in differential expression analyses, ranged from 29 to 57% (Table 3) and specificity ranged from 64 to 100%. Using a baseline IgE cutoff of ≥ 100 IU/mL had a sensitivity of 57% and specificity of 64%. Its specificity increased when combined with CXCL10 or its ratios with a worsening of its sensitivity. The ratio of CXCL10 to CCL17 had the highest sensitivity of 71% and had a specificity of 79% (Table 3 and Supplemental Figure S6).

**Table 3:** Sensitivity, Specificity, and Accuracy of predicting omalizumab response using cutoffs defined by the top cytokine or ratios.

*Measures used in cutoff	Sensitivity	Specificity	Accuracy	AUC
Optimal CXCL10	0.57	1.00	0.86	0.63
Optimal CXCL10/IL-21	0.57	0.93	0.81	0.65
Optimal CXCL10/CCL17	0.71	0.79	0.76	0.77
IgE ≥ 100 + CXCL10	0.43	1.00	0.81	0.66
Optimal CXCL10/IL-13	0.43	1.00	0.81	0.64
Optimal CXCL10/IL-4	0.43	1.00	0.81	0.59
Optimal CXCL10/IL-17B	0.43	1.00	0.81	0.61
IgE ≥ 100 + CXCL10/IL-21	0.43	0.93	0.76	0.66
IgE ≥ 100 + CXCL10/IL-13	0.29	1.00	0.76	0.66
IgE ≥ 100 + CXCL10/IL-4	0.29	1.00	0.76	0.62
IgE ≥ 100 + CXCL10/IL-17B	0.29	1.00	0.76	0.64
IgE ≥ 100	0.57	0.64	0.62	0.61
IgE ≥ 100 + CXCL10/CCL17	0.29	0.93	0.71	0.62

*IgE: total immunoglobulin E in ku/L at baseline; ‘Optimal’: using the cut-off point for the cytokine or ratio that maximizes predictive accuracy.

## Discussion

In this relatively small cohort of patients with severe asthma receiving mepolizumab or omalizumab, baseline plasma IL-13 levels were differentially expressed and higher in patients who subsequently had a clinical response to mepolizumab, while plasma CXCL10 levels were higher in responders to omalizumab. Baseline levels of IL-5, the target of mepolizumab, also tended to be higher in mepolizumab responders but did not significantly differentiate responders from nonresponders in this cohort of patients with severe eosinophilic asthma patients with high burden of exacerbations at baseline (average exacerbations and eosinophil count: 3 exacerbations in the prior year and 244 eosinophils per µL). In descriptive analyses, we found multiple relationships between pairs of cytokines to be different pretreatment in responders compared to nonresponders. In general, responders tended to have stronger negative correlations between Th2-type cytokines and other pro-inflammatory, Th1-, or Th-17-type cytokines. For mepolizumab, responders were more likely to demonstrate strong negative correlations between a Th2-attracting chemokine or alarmin (TSLP, CCL22) and a Th-17 associated cytokine (IL-17A, IL-17C, IL-23) while these relationships were positively correlated or poorly negatively correlated in non-responders. This might reflect patients that have clearly T2-high asthma, rather than a mixed endotype, and thus likely to respond better to mepolizumab. Similarly, for omalizumab, many of the inversed relationships at baseline between responders and nonresponders involved Th2-type cytokines and Th1-type cytokines with responders having stronger negative correlations between Th1 and Th2 type cytokines and chemokines, in general, compared to nonresponders in whom there was a single strong negative correlation which was between IL-17F and IL-6.

Mepolizumab is approved for the treatment of severe eosinophilic asthma, a “type 2” inflammatory-associated phenotype. IL-13, which we found to be significantly associated with response, induces type 2 inflammation and promotes eosinophilic airway inflammation^35^. It regulates eotaxin-dependent eosinophil tracking to the lungs in allergen-mediated airway inflammation^36^ and upregulates CCL22 levels^37^. CCL22 has been shown to induce airway eosinophil migration in both mouse models and humans ^38,39^. Thus, although all mepolizumab-treated patients in this study met criteria for eosinophilic asthma based on their pre-treatment peripheral blood eosinophil counts, peripheral eosinophilia does not necessarily correlate with airway eosinophilia and patients with higher IL-13 levels at baseline may have higher airway tissue eosinophilia and be more likely to respond to anti-eosinophil agents like mepolizumab. It is also not surprising that in this cohort, IL-13 was more predictive of response compared to IL-5, mepolizumab’s target and a cytokine important for eosinophil survival and maturation, since mepolizumab patients are preselected for having high peripheral eosinophil counts thus limiting the variability in IL-5 levels. However, another study using sputum levels of inflammatory markers found that only anti-eosinophil peroxidase (EPX) antibody levels, but not IL-5 or IL-13 levels, were associated with suboptimal response to mepolizumab in asthma^40^. This is consistent with prior studies using sputa showing that many patients with severe asthma have autoantibodies in their airways which may correlate with poor response to therapies^41,42^.

For omalizumab, our finding that higher levels of CXCL10 was associated with better response is consistent with prior studies that have shown that elevated CXCL10 is associated with omalizumab response and/or promotes allergic airway inflammation.^43–45^ Elevated CXCL10 might reflect a group of patients who meet eligibility criteria for omalizumab and whose asthma is predominantly triggered by environmental allergens, as opposed to patients who may have perennial rhinitis but whose asthma is primarily triggered by other agents like smoke^10,44^. Additionally, although these patients had on average two exacerbations in the year prior to omalizumab, elevated levels of CXCL10 may correlate further with asthma severity suggesting a subcohort of patients with a higher burden of exacerbations as shown in a prior study^46^. Multiple factors are associated with nonresponse to biologics including the existence of ongoing airway autoimmune pathology^47^. CXCL10 levels are elevated in a wide range of autoimmune diseases and, although we found that it is elevated in responders, is a pleotropic cytokine in need of additional research on its role in response to omalizumab.

We also found the IL-13/ TNF-α ratio and the ratio of CXCL10 to CCL17 improve the predictive accuracy of IL-13 and CXCL10 in predicting response to mepolizumab and omalizumab respectively. These patients may represent patients with distinct (high ratios) versus mixed endotypes (low ratios). Asthma is a highly heterogeneous disease and there is ample evidence that the classification of asthma into T2-high versus T2-low is highly simplistic with the existence of multiple subendotypes and subphenotypes^48,49^. Patients with severe asthma demonstrate even higher phenotypic and endotypic heterogeneity characterized by a complex interaction of the Th1, Th2, and Th17 axes.^49–51^ Pro-inflammatory, Th1, and Th17 cytokines including IFN-γ, TNF-α, IL-17A, IL-17F, and IL-22 are associated with severe steroid-resistant asthma and neutrophilic inflammation.^52–54^ Higher baseline levels of the IL-13/TNF-α ratio, which we found to have the best sensitivity and specificity in predicting mepolizumab response, may indicate patients who have a predominantly eosinophilic endotype rather than a mixed eosinophilic and neutrophilic endotype thus demonstrating better response to mepolizumab. In a prior study, TNF-α, a neutrophil and eosinophil chemoattractant, increased sputum neutrophilia and airway hyperresponsiveness in normal subjects.^55^ TNF-α also promotes the damaging effects of eosinophilic inflammation on the epithelium.^52^ Thus, elevations of both IL-13 and TNF-α may suggest a ‘double hit’ in these mepolizumab patients. In two previous studies, 10 to 12 weeks of etanercept, an anti-TNF fusion protein, improved lung function, bronchial responsiveness, and asthma-related quality of life^56,57^. This has however not been replicated in other studies likely due to differences in the study populations and suggesting that only patients with T2 high asthma and a hyperactive TNF axis may benefit from TNF-targeted therapy^52,58,59^. Similarly, for omalizumab, a higher ratio of CXCL10 to CCL17 and to the Th-17 cytokine, IL-21, were associated with better response to omalizumab. This may suggest patients whose asthma pathology is predominantly allergic rather than those with other autoinflammatory or Th-17 driven disease which, not surprisingly, would not respond as well to the anti-IgE agent, omalizumab. However, there is also prior evidence that up to one-third to half of patients with severe asthma may have a type 1 inflammatory signature in their airways which is associated with more severe disease and corticosteroid resistance and potentially involving the interferon-gamma/CXCL10 axis^60,61^. Thus, additional research is needed into the implications of these findings.

Our results should be interpreted with caution. First, the cohort was relatively small. Thus, we had limited statistical power and did not adjust for all possible confounders and multiple testing. Although our findings are biologically plausible, they are novel and need to be validated in larger and more diverse cohorts. Relatedly, most of our analyses should be seen as exploratory, not confirmatory, and the associations we have found may not be causal. For instance, the correlations between protein pairs are descriptive and do not indicate causality^62^. Secondly, we may have missed exacerbations treated at centers outside the MGB health system. However, this is likely to be an infrequent occurrence since most patients receiving their respiratory biologics in the MGB system will be more likely to be followed within the system. Thirdly, we conducted proteomic assay only on baseline plasma samples. Thus, we were unable to assess how the biologics affected the levels of these cytokines over time or to evaluate how changes in medication dosage might have impacted proteins or outcomes. Though our goal was to establish biomarkers that providers can use when selecting a respiratory biologic, future studies should evaluate longitudinal changes in protein levels and in medication doses and how these changes impact therapeutic response. Also, there was a lag between sample collection and therapy initiation in many patients. However, studies have shown great intra-person stability of the proteome, even in asthma patients, and we had limited the samples to those collected at the patient’s steady state^63,64^. Furthermore, the top cytokines remained the same in sensitivity analyses including this lag in differential analyses. Finally, the proteomic assay evaluated relative quantities, not absolute, of proteins. Thus, the values of these proteins would likely differ in a different population. However, these relative quantifications are a valid measure of these proteins in our cohort^23^.

In this study, IL-13 differentiated mepolizumab responders from non-responders and CXCL10 differentiated omalizumab responders from nonresponders. The relationships between the pretreatment levels of many cytokines differed between patients who responded and those who did not respond to mepolizumab or omalizumab. The ratios of IL-13 and CXCL10 to multiple Th-1 and Th-17 cytokines and chemokines were associated with response to mepolizumab and omalizumab respectively. These findings are biologically meaningful and considering the high clinical and financial importance of identifying biomarkers predictive of response to these respiratory biologics, the study findings should be explored in further studies.

### Supplementary Information

Supplementary Information.

## Data Availability

Data related to this study will be available upon request from potential collaborators and upon execution of a data use agreement approved by the Mass General Brigham IRB.

## References

[1] Brusselle GG, Koppelman GH (2022). Biologic therapies for severe asthma. N. Engl. J. Med..

[2] Akenroye, A.*, et al.* Comparative efficacy of mepolizumab, benralizumab, and dupilumab in eosinophilic asthma: A Bayesian network meta-analysis. *J. Allergy Clin. Immunol.* (2022). 10.1016/j.jaci.2022.05.024 PMC9643621 35772597

[3] Akenroye AT (2023). Comparative effectiveness of omalizumab, mepolizumab, and dupilumab in asthma: A target trial emulation. J. Allergy Clin. Immunol..

[4] Akenroye A, McCormack M, Keet C (2020). Severe asthma in the US population and eligibility for mAb therapy. J. Allergy Clin. Immunol..

[5] Akenroye, A.*, et al.* Switch patterns in a cohort of individuals with asthma who received omalizumab or mepolizumab therapy. *J. Allergy Clin. Immunol.* (2022). 10.1016/j.jaip.2022.10.047 PMC10006368 36384205

[6] Akenroye, A.*, et al.* Incidence of adverse events prompting switching between biologics among adults with asthma: A retrospective cohort study. *Allergy* (2022). 10.1111/all.15564 PMC10066822 36286487

[7] Mauger D, Apter AJ (2019). Indirect treatment comparisons and biologics. J. Allergy Clin. Immunol..

[8] Albers FC (2019). Baseline blood eosinophil count as a predictor of treatment response to the licensed dose of mepolizumab in severe eosinophilic asthma. Respir. Med..

[9] Dupilumab in moderate-to-severe atopic dermatitis with or without comorbid asthma: pooled analysis of 2 randomized phase 3 trials (LIBERTY AD SOLO 1 & 2). *Journal of allergy and clinical immunology* Conference: 2018 American Academy of Allergy, Asthma and Immunology, AAAAI and World Allergy Organization, WAO Joint Congress. United States. 141, AB132 (2018).

[10] Medoff BD (2002). IFN-gamma-inducible protein 10 (CXCL10) contributes to airway hyperreactivity and airway inflammation in a mouse model of asthma. J. Immunol..

[11] Movérare R, Elfman L, Stålenheim G, Björnsson E (2000). Study of the Th1/Th2 balance, including IL-10 production, in cultures of peripheral blood mononuclear cells from birch-pollen-allergic patients. Allergy.

[12] Rastogi D (2015). Inflammation, metabolic dysregulation, and pulmonary function among obese urban adolescents with asthma. Am. J. Respir. Crit. Care Med..

[13] Zoratti E (2014). Differentiating asthma phenotypes in young adults through polyclonal cytokine profiles. Annals Allergy Asthma Immunol. Off. Publ. Am. College Allergy Asthma Immunol..

[14] Ali, K., Wu, L., Qiu, Y. & Li, M. Case report: Clinical and histopathological characteristics of psoriasiform erythema and de novo IL-17A cytokines expression on lesioned skin in atopic dermatitis children treated with dupilumab. *Front. Med.* **9**, 932766 (2022). 10.3389/fmed.2022.932766 PMC9366075 35966849

[15] Hu C (2018). Glucocorticoids modulate Th1 and Th2 responses in asthmatic mouse models by inhibition of notch1 signaling. Int. Arch. Allergy Immunol..

[16] Huang JL (2003). TH1 and TH2 cytokine production among asthmatic children after immunotherapy. J. Asthma Off. J. Assoc. Care Asthma.

[17] Kuo ML, Huang JL, Yeh KW, Li PS, Hsieh KH (2001). Evaluation of Th1/Th2 ratio and cytokine production profile during acute exacerbation and convalescence in asthmatic children. Annals Allergy Asthma Immunol. Off. Publ. Am. College Allergy Asthma Immunol..

[18] Zijlstra GJ (2014). Glucocorticoids induce the production of the chemoattractant CCL20 in airway epithelium. Eur. Respir. J..

[19] Castro VM (2022). The mass general brigham biobank portal: An i2b2-based data repository linking disparate and high-dimensional patient data to support multimodal analytics. J. Am. Med. Inform. Assoc..

[20] Busse, W.W., Morgan, W.J., Taggart, V. & Togias, A. Asthma outcomes workshop: overview. *J. Allerg. Clin. Immunol.* 129 S1–8 (Published by Mosby, Inc., 2012). 10.1016/j.jaci.2011.12.985 PMC4259286 22386504

[21] Foer D (2021). Asthma exacerbations in patients with type 2 diabetes and asthma on glucagon-like peptide-1 receptor agonists. Am. J. Respir. Crit. Care Med..

[22] General Assembly of the World Medical Association (2014). World medical association declaration of Helsinki: Ethical principles for medical research involving human subjects. J. Am. Coll. Dent..

[23] Candia J (2017). Assessment of variability in the SOMAscan assay. Sci. Rep..

[24] Pietzner M (2021). Synergistic insights into human health from aptamer- and antibody-based proteomic profiling. Nat. Commun..

[25] Mitchell PD, O'Byrne PM (2017). Epithelial-derived cytokines in asthma. Chest.

[26] Boonpiyathad T, Sözener ZC, Satitsuksanoa P, Akdis CA (2019). Immunologic mechanisms in asthma. Semin Immunol.

[27] Guglani L, Khader SA (2010). Th17 cytokines in mucosal immunity and inflammation. Curr Opin HIV AIDS.

[28] Halwani R (2017). Th-17 regulatory cytokines IL-21, IL-23, and IL-6 enhance neutrophil production of IL-17 cytokines during asthma. J. Asthma Off. J. Assoc. Care Asthma.

[29] Lai ST (2008). T-helper 1-related chemokines in the exacerbation of childhood asthma. Pediatr. Int..

[30] Zhao Y, Yang J, Gao YD (2011). Altered expressions of helper T cell (Th)1, Th2, and Th17 cytokines in CD8(+) and γδ T cells in patients with allergic asthma. J. Asthma Off. J. Assoc. Care Asthma.

[31] Akoglu H (2018). User's guide to correlation coefficients. Turk. J. Emerg. Med..

[32] Ritchie ME (2015). limma powers differential expression analyses for RNA-sequencing and microarray studies. Nucleic Acids Res.

[33] Busse W (2019). Anti-IL-5 treatments in patients with severe asthma by blood eosinophil thresholds: Indirect treatment comparison. J. Allergy Clin. Immunol..

[34] Sheehan, W.J.*, et al.* Aeroallergen sensitization, serum IgE, and eosinophilia as predictors of response to omalizumab therapy during the fall season among children with persistent asthma. *J. Allergy Clin. Immunol.* **8**, 3021–3028 (2020). 10.1016/j.jaip.2020.03.051 PMC8775809 32376491

[35] Doran, E.*, et al.* Interleukin-13 in asthma and other eosinophilic disorders. *Front Med* **4**, 139 (2017). 10.3389/fmed.2017.00139 PMC5627038 29034234

[36] Gazzinelli-Guimaraes PH (2023). Eosinophil trafficking in allergen-mediated pulmonary inflammation relies on IL-13-driven CCL-11 and CCL-24 production by tissue fibroblasts and myeloid cells. J. Allergy Clin. Immunol. Glob..

[37] Yamashita U, Kuroda E (2002). Regulation of macrophage-derived chemokine (MDC, CCL22) production. Crit. Rev. Immunol..

[38] Pinho V (2003). The role of CCL22 (MDC) for the recruitment of eosinophils during allergic pleurisy in mice. J. Leukoc. Biol..

[39] Liu LY, Jarjour NN, Busse WW, Kelly EA (2003). Chemokine receptor expression on human eosinophils from peripheral blood and bronchoalveolar lavage fluid after segmental antigen challenge. J. Allergy Clin. Immunol..

[40] Mukherjee, M.*, et al.* Suboptimal treatment response to anti-IL-5 monoclonal antibodies in severe eosinophilic asthmatics with airway autoimmune phenomena. *The European respiratory journal* **56**(2020). 10.1183/13993003.00117-2020 32444405

[41] Mukherjee M (2018). Sputum autoantibodies in patients with severe eosinophilic asthma. J. Allergy Clin. Immunol..

[42] Salter, B.*, et al.* Airway autoantibodies are determinants of asthma severity. *European Respirat. J. ***60**(2022). 10.1183/13993003.00442-2022 35777765

[43] Suzukawa M (2018). Baseline serum CXCL10 and IL-12 levels may predict severe asthmatics' responsiveness to omalizumab. Respir. Med..

[44] Alrashdan YA (2012). Asthmatic airway smooth muscle CXCL10 production: Mitogen-activated protein kinase JNK involvement. Am. J. Physiol. Lung Cell Mol. Physiol..

[45] Huoman J (2021). Childhood CCL18, CXCL10 and CXCL11 levels differentially relate to and predict allergy development. Pediatr. Allergy Immunol..

[46] Osman HM, El Basha NR, Mansour AF, Hanna MOF (2022). Serum IFNγ-induced protein 10 (IP10/CXCL10): Association with asthma exacerbations and severity in children. J. Asthma Off. J. Assoc. Care Asthma.

[47] Salter B, Lacy P, Mukherjee M (2021). Biologics in asthma: A molecular perspective to precision medicine. Front. Pharmacol..

[48] Fajt, M.L. & Wenzel, S.E. Asthma phenotypes and the use of biologic medications in asthma and allergic disease: the next steps toward personalized care. *J. Allergy Clin. Immunol. ***135**, 299–310 (2015). 10.1016/j.jaci.2014.12.1871 25662302

[49] Wenzel SE (2006). Asthma: Defining of the persistent adult phenotypes. Lancet.

[50] Green RH, Brightling CE, Bradding P (2007). The reclassification of asthma based on subphenotypes. Curr. Opin. Allergy Clin. Immunol..

[51] Bradding P, Green RH (2010). Subclinical phenotypes of asthma. Curr. Opin. Allergy Clin. Immunol..

[52] Brightling, C., Berry, M. & Amrani, Y. Targeting TNF-alpha: a novel therapeutic approach for asthma. *J. Allerg. Clin. Immunol*. **121**:11–12 (2008). 10.1016/j.jaci.2007.10.028 PMC3992375 18036647

[53] Newcomb DC, Peebles RS (2013). Th17-mediated inflammation in asthma. Curr. Opin. Immunol..

[54] Luo W, Hu J, Xu W, Dong J (2022). Distinct spatial and temporal roles for Th1, Th2, and Th17 cells in asthma. Front. Immunol..

[55] Thomas PS, Yates DH, Barnes PJ (1995). Tumor necrosis factor-alpha increases airway responsiveness and sputum neutrophilia in normal human subjects. Am. J. Respir. Crit. Care. Med..

[56] Howarth PH (2005). Tumour necrosis factor (TNFalpha) as a novel therapeutic target in symptomatic corticosteroid dependent asthma. Thorax.

[57] Berry MA (2006). Evidence of a role of tumor necrosis factor alpha in refractory asthma. N. Engl. J. Med..

[58] Rouhani FN (2005). Effect of tumor necrosis factor antagonism on allergen-mediated asthmatic airway inflammation. Respir. Med..

[59] Morjaria JB (2008). The role of a soluble TNFalpha receptor fusion protein (etanercept) in corticosteroid refractory asthma: a double blind, randomised, placebo controlled trial. Thorax.

[60] Gauthier M (2023). CCL5 is a potential bridge between type 1 and type 2 inflammation in asthma. J. Allergy Clin. Immunol..

[61] Gauthier, M.*, et al.* Severe asthma in humans and mouse model suggests a CXCL10 signature underlies corticosteroid-resistant Th1 bias. *JCI Insight* **2**(2017). 10.1172/jci.insight.94580 PMC5499373 28679952

[62] Schober P, Boer C, Schwarte LA (2018). Correlation coefficients: Appropriate use and interpretation. Anesth Analg.

[63] Kim CH (2018). Stability and reproducibility of proteomic profiles measured with an aptamer-based platform. Sci Rep.

[64] Asamoah K (2024). Proteomic signatures of eosinophilic and neutrophilic asthma from serum and sputum. EBioMedicine.

