# Supplementary material for: Ratio of plasma IL-13/TNF- ∝ and CXCL10/CCL17 predicts mepolizumab and omalizumab response in asthma better than eosinophil count or immunoglobulin E level
Source: Sci Rep. 2024 May 6;14:10404. doi: 10.1038/s41598-024-60864-3 (PMC11074109; doi:10.1038/s41598-024-60864-3)
Supplement: Supplementary file 1 — Supplementary Information. [file 41598_2024_60864_MOESM1_ESM.docx]

**SUPPLEMENTARY TABLES AND FIGURES**

| **Table S1: Baseline characteristics of participants by response status** | | | | |
| --- | --- | --- | --- | --- |
|  | **Mepolizumab** | | **Omalizumab** | |
|  | **Responder** | **Non-responder** | **Responder** | **Non-responder** |
| N | 7 | 11 | 7 | 14 |
| Age in years, mean (SD) | 58.9 (9.4) | 54.0 (13.5) | 46.4 (19.2) | 44.6 (13.6) |
| Female, n (%) | 3 (43) | 10 (91) | 6 (86) | 12 (86) |
| White race, n (%) | 4 (57) | 9 (82) | 5 (71) | 14 (100) |
| Current smoker, n (%) | 0 (0) | 1 (9) | 0 (0) | 0 (0) |
| Former smoker, n (%) | 0 (0) | 5 (46) | 2 (29) | 1 (7) |
| BMI, kg/m^2^; mean (SD) | 32.3 (7.3) | 29.4 (7.4) | 30.5 (7.8) | 28.8 (8.3) |
| Baseline annualized exacerbation rate, mean (SD) | 3.6 (1.5) | 2.6 (2.0) | 2.7 (1.7) | 1.9 (1.5) |
| Baseline pre-bronchodilator FEV1, L; mean (SD)^a^ | 2.41 (0.87) | 1.82 (0.81) | 2.46 (1.11) | 2.55 (0.77) |
| Baseline pre-bronchodilator FEV1 % predicted, mean (SD)^a^ | 81.6 (10.8) | 65.0 (25.5) | 78.0 (25.2) | 89.3 (17.8) |
| Peripheral blood eosinophil counts, cells/µL; median [IQR]^b^ |  |  |  |  |
| *At mepolizumab initiation* | 362 [291-391] | 140 [27-269] | 152 [89-247] | 100 [67-300] |
| *Maximum count within 3 years of mepolizumab initiation* | 480 [391-650] | 600 [225-1710] | 152 [95-247] | 239 [93-644] |
| Immunoglobulin E (IgE), IU/µL; median [IQR]^b^ | 332 [241-448] | 96 [43-546] | 185 [61-257] | 77 [34-256] |
| Allergic rhinitis, n (%) | 7 (100) | 8 (73) | 7 (100) | 14 (100) |
| Atopic dermatitis, n (%) | 0 (0) | 0 (0) | 0 (0) | 0 (0) |
| COPD, n (%) | 0 (0) | 1 (9) | 0 (0) | 0 (0) |
| BMI, Body mass index; COPD, chronic obstructive pulmonary disease; FEV1, forced expiratory volume in one second; IQR, interquartile range; OCS, oral corticosteroids; SD, standard deviation | | | | |
| ^a^Three omalizumab non-responders and two responders were missing baseline pulmonary function test and two mepolizumab responders with missing baseline pulmonary function test.  ^b^One omalizumab non-responder was missing baseline blood eosinophil counts and one mepolizumab responder with missing baseline IgE. | | | | |
|  | | |  |  |

| **Table S2: Adjusted differential expression analysis (limma) results for mepolizumab response** | | | | | | | |
| --- | --- | --- | --- | --- | --- | --- | --- |
| **Cytokine** | **Abbreviation** | **EntrezGeneSymbol** | **logFC** | **95% CI (lower bound)** | **95% CI  (upper bound)** | **Average Expression** | ***P-value** |
| Interleukin-13 | IL-13 | IL13 | 0.425 | 0.021 | 0.828 | 9.934 | 0.040 |
| Tumor necrosis factor | TNF-a | TNF | -0.775 | -1.994 | 0.444 | 9.201 | 0.197 |
| Interleukin-5 | IL-5 | IL5 | 0.278 | -0.181 | 0.738 | 11.111 | 0.218 |
| Growth-regulated alpha protein | Gro-a | CXCL1 | 0.504 | -0.341 | 1.348 | 10.582 | 0.225 |
| C-X-C motif chemokine 10 | IP-10 | CXCL10 | 0.256 | -0.192 | 0.705 | 9.459 | 0.245 |
| Interleukin-17B | IL-17B | IL17B | 0.168 | -0.145 | 0.482 | 8.579 | 0.274 |
| C-C motif chemokine 1 | I-309 | CCL1 | -0.211 | -0.617 | 0.195 | 10.031 | 0.288 |
| Interferon gamma | IFN-g | IFNG | 0.158 | -0.195 | 0.511 | 8.712 | 0.358 |
| Interleukin-22 | IL-22 | IL22 | -0.191 | -0.633 | 0.250 | 9.540 | 0.373 |
| Interleukin-1 alpha | IL-1a | IL1A | -0.080 | -0.291 | 0.130 | 9.120 | 0.432 |
| Interleukin-25 | IL-17E | IL25 | -0.146 | -0.554 | 0.263 | 8.218 | 0.463 |
| Interleukin-17A | IL-17 | IL17A | 0.185 | -0.342 | 0.713 | 8.221 | 0.468 |
| Interleukin-6 | IL-6 | IL6 | -0.142 | -0.572 | 0.288 | 8.467 | 0.495 |
| Interleukin-8 | IL-8 | CXCL8 | 0.068 | -0.148 | 0.284 | 9.567 | 0.514 |
| C-C motif chemokine 22 | MDC | CCL22 | 0.171 | -0.410 | 0.752 | 10.378 | 0.543 |
| Interleukin-4 | IL-4 | IL4 | -0.174 | -0.769 | 0.421 | 8.896 | 0.545 |
| Interleukin-17F | IL-17F | IL17F | -0.037 | -0.167 | 0.092 | 9.700 | 0.550 |
| Thymic stromal lymphopoietin | TSLP | TSLP | 0.063 | -0.182 | 0.308 | 8.604 | 0.592 |
| Interleukin-21 | IL-21 | IL21 | 0.089 | -0.297 | 0.475 | 10.176 | 0.633 |
| Interleukin-17C | IL-17C | IL17C | -0.039 | -0.305 | 0.227 | 10.218 | 0.761 |
| C-C motif chemokine 17 | TARC | CCL17 | -0.056 | -0.594 | 0.482 | 8.817 | 0.829 |
| Interleukin-1 beta | IL-1b | IL1B | 0.029 | -0.294 | 0.352 | 10.498 | 0.851 |
| Interleukin-17D | IL-17D | IL17D | 0.032 | -0.420 | 0.484 | 8.390 | 0.883 |
| Interleukin-23 | IL-23 | IL12B\|IL23A | 0.017 | -0.395 | 0.429 | 9.556 | 0.933 |
| Interleukin-10 | IL-10 | IL10 | 0.005 | -0.209 | 0.219 | 7.515 | 0.961 |
| Interleukin-9 | IL-9 | IL9 | -0.007 | -0.333 | 0.319 | 8.036 | 0.966 |
| CI: Confidence interval |  |  |  |  |  |  |  |
| *P-value is not corrected for multiple testing | |  |  |  |  |  |  |

| **Table S3: Adjusted differential expression analysis (limma) results for top 20 cytokine/chemokine ratios associated with mepolizumab response** | | | | | |
| --- | --- | --- | --- | --- | --- |
| **Cytokine ratio** | **Log Fold Change** | **95% CI (lower bound)** | **95% CI  (upper bound)** | **Average Expression** | ***P-value** |
| IL-13/CCL1 | 0.061 | 0.008 | 0.114 | 0.991 | 0.027 |
| IL-13/IL-1a | 0.056 | 0.006 | 0.106 | 1.090 | 0.031 |
| IL-13/IL-25 | 0.072 | 0.007 | 0.137 | 1.211 | 0.032 |
| IL-13/TNF-a | 0.111 | 0.008 | 0.214 | 1.093 | 0.036 |
| IL-13/IL-17F | 0.048 | 0.001 | 0.094 | 1.024 | 0.044 |
| IL-13/IL-22 | 0.063 | -0.001 | 0.126 | 1.043 | 0.053 |
| IL-13/IL-17C | 0.045 | -0.002 | 0.092 | 0.973 | 0.059 |
| IP-10/IL-6 | 0.049 | -0.004 | 0.103 | 1.119 | 0.069 |
| TNF-a/CCL22 | -0.084 | -0.179 | 0.011 | 0.887 | 0.081 |
| TNF-a/CXCL1 | -0.104 | -0.224 | 0.016 | 0.874 | 0.085 |
| IL-13/IL-6 | 0.071 | -0.012 | 0.153 | 1.177 | 0.088 |
| IL-13/IL-4 | 0.065 | -0.011 | 0.142 | 1.121 | 0.091 |
| IL-13/IL-10 | 0.057 | -0.011 | 0.124 | 1.323 | 0.095 |
| TNF-a/CXCL10 | -0.113 | -0.249 | 0.023 | 0.977 | 0.097 |
| IFN-g/IL-25 | 0.038 | -0.008 | 0.084 | 1.061 | 0.100 |
| TNF-a/IL-17B | -0.108 | -0.242 | 0.026 | 1.073 | 0.107 |
| IL-13/IL-1b | 0.037 | -0.009 | 0.084 | 0.947 | 0.112 |
| IL-5/CCL1 | 0.048 | -0.013 | 0.110 | 1.109 | 0.113 |
| TNF-a/IL-5 | -0.089 | -0.203 | 0.025 | 0.830 | 0.117 |
| TNF-a/IFN-g | -0.109 | -0.249 | 0.031 | 1.058 | 0.118 |
| CI: Confidence interval | | | | | |
| *P-value is not corrected for multiple testing | | | | | |

| **Table S4: Adjusted differential expression analysis (limma) results for omalizumab response** | | | | | | | |
| --- | --- | --- | --- | --- | --- | --- | --- |
| **Cytokine** | **Abbreviation** | **EntrezGeneSymbol** | **logFC** | **95% CI (lower bound)** | **95% CI  (upper bound)** | **Average Expression** | ***P-value** |
| C-X-C motif chemokine 10 | IP-10 | CXCL10 | 0.926 | 0.173 | 1.680 | 9.550 | 0.018 |
| Interleukin-21 | IL-21 | IL21 | -0.302 | -0.615 | 0.011 | 10.073 | 0.058 |
| Interleukin-25 | IL-17E | IL25 | 0.812 | -0.055 | 1.680 | 8.355 | 0.065 |
| Interleukin-17D | IL-17D | IL17D | 0.352 | -0.079 | 0.783 | 8.283 | 0.104 |
| Growth-regulated alpha protein | Gro-a | CXCL1 | 0.398 | -0.161 | 0.957 | 10.801 | 0.153 |
| Interleukin-23 | IL-23 | IL12B\|IL23A | 0.266 | -0.113 | 0.645 | 9.766 | 0.159 |
| Interleukin-13 | IL-13 | IL13 | -0.189 | -0.507 | 0.128 | 9.715 | 0.228 |
| Interleukin-1 beta | IL-1b | IL1B | 0.260 | -0.194 | 0.715 | 10.410 | 0.246 |
| Interleukin-17A | IL-17 | IL17A | 0.147 | -0.189 | 0.483 | 8.184 | 0.374 |
| C-C motif chemokine 22 | MDC | CCL22 | 0.279 | -0.379 | 0.937 | 10.469 | 0.387 |
| Interleukin-6 | IL-6 | IL6 | 0.156 | -0.219 | 0.532 | 8.508 | 0.396 |
| Tumor necrosis factor | TNF-a | TNF | -0.068 | -0.241 | 0.104 | 8.794 | 0.420 |
| Interleukin-10 | IL-10 | IL10 | 0.113 | -0.182 | 0.408 | 7.454 | 0.436 |
| C-C motif chemokine 1 | I-309 | CCL1 | -0.070 | -0.257 | 0.117 | 9.925 | 0.443 |
| Interleukin-5 | IL-5 | IL5 | 0.090 | -0.174 | 0.355 | 11.043 | 0.485 |
| C-C motif chemokine 17 | TARC | CCL17 | 0.169 | -0.358 | 0.695 | 8.858 | 0.511 |
| Interleukin-17B | IL-17B | IL17B | -0.141 | -0.685 | 0.404 | 8.549 | 0.596 |
| Interleukin-1 alpha | IL-1a | IL1A | 0.128 | -0.533 | 0.790 | 9.337 | 0.690 |
| Interleukin-8 | IL-8 | CXCL8 | 0.047 | -0.216 | 0.310 | 9.588 | 0.712 |
| Interleukin-22 | IL-22 | IL22 | -0.123 | -0.875 | 0.629 | 9.609 | 0.737 |
| Interleukin-17C | IL-17C | IL17C | 0.048 | -0.260 | 0.357 | 10.217 | 0.749 |
| Interleukin-17F | IL-17F | IL17F | 0.030 | -0.168 | 0.229 | 9.768 | 0.753 |
| Interferon gamma | IFN-g | IFNG | 0.029 | -0.217 | 0.275 | 8.595 | 0.810 |
| Thymic stromal lymphopoietin | TSLP | TSLP | 0.022 | -0.246 | 0.290 | 8.637 | 0.865 |
| Interleukin-9 | IL-9 | IL9 | 0.019 | -0.211 | 0.248 | 7.813 | 0.868 |
| Interleukin-4 | IL-4 | IL4 | -0.002 | -0.262 | 0.258 | 8.825 | 0.988 |
| CI: Confidence interval |  |  |  |  |  |  |  |
| *P-value is not corrected for multiple testing | |  |  |  |  |  |  |

| **Table S5: Adjusted differential expression analysis (limma) results for top 20 cytokine/chemokine ratios associated with omalizumab response** | | | | | |
| --- | --- | --- | --- | --- | --- |
| **Cytokine ratio** | **Log Fold Change** | **95% CI (lower bound)** | **95% CI  (upper bound)** | **Average Expression** | ***P-value** |
| CXCL10/IL-21 | 0.126 | 0.036 | 0.215 | 0.950 | 0.008 |
| CXCL10/IL-13 | 0.116 | 0.030 | 0.201 | 0.985 | 0.010 |
| CXCL10/CCL17 | 0.077 | 0.020 | 0.135 | 1.078 | 0.010 |
| CXCL10/IL-4 | 0.106 | 0.024 | 0.188 | 1.083 | 0.013 |
| CXCL10/IL-17B | 0.123 | 0.027 | 0.218 | 1.120 | 0.014 |
| CXCL10/IL-17F | 0.090 | 0.018 | 0.162 | 0.978 | 0.016 |
| CXCL10/TSLP | 0.104 | 0.021 | 0.188 | 1.106 | 0.017 |
| CXCL10/IL-10 | 0.101 | 0.019 | 0.182 | 1.281 | 0.018 |
| CXCL10/TNF-a | 0.117 | 0.021 | 0.213 | 1.087 | 0.019 |
| CXCL10/CCL1 | 0.103 | 0.017 | 0.189 | 0.963 | 0.021 |
| CXCL10/IL-8 | 0.092 | 0.014 | 0.169 | 0.996 | 0.023 |
| IL-21/IL-23 | -0.058 | -0.107 | -0.008 | 1.033 | 0.024 |
| IL-21/IL-25 | -0.118 | -0.219 | -0.016 | 1.218 | 0.025 |
| IL-21/IL-17D | -0.080 | -0.149 | -0.010 | 1.220 | 0.026 |
| CXCL10/IL-9 | 0.116 | 0.015 | 0.217 | 1.223 | 0.027 |
| CXCL10/IL-5 | 0.076 | 0.008 | 0.144 | 0.865 | 0.029 |
| CXCL10/IL-17A | 0.089 | 0.010 | 0.168 | 1.167 | 0.030 |
| CXCL10/IL-6 | 0.085 | 0.009 | 0.162 | 1.122 | 0.031 |
| IL-25/IL-17A | 0.073 | 0.007 | 0.140 | 1.019 | 0.031 |
| CXCL10/IL-17C | 0.086 | 0.008 | 0.164 | 0.935 | 0.033 |
| CI: Confidence interval | | | | | |
| *P-value is not corrected for multiple testing | | | | | |


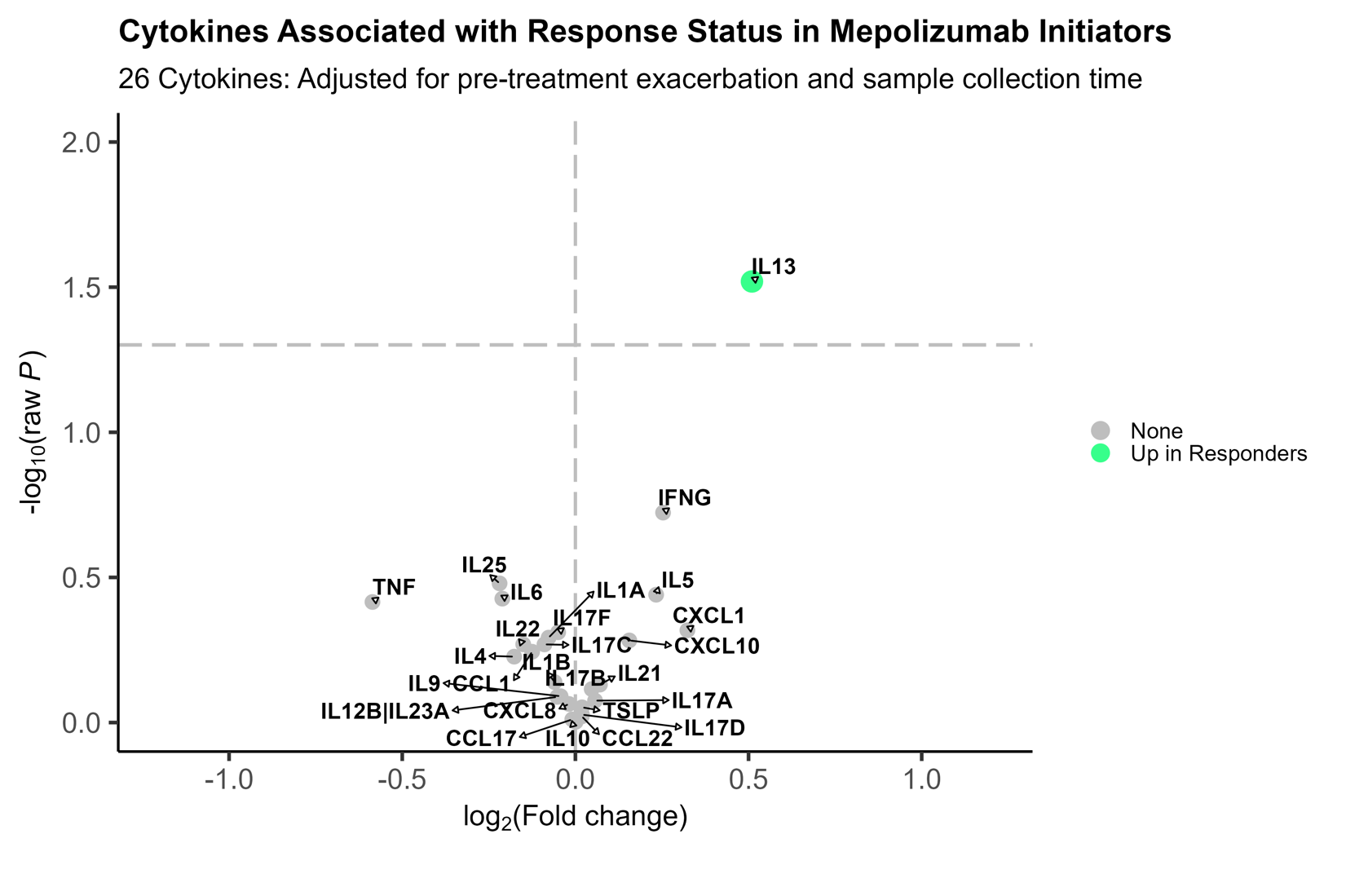


**Figure S1. Differential expression analysis for 26 cytokines and response status in the mepolizumab group** **adjusted for pre-treatment annualized exacerbation rate and time between sample collection and initiation of mepolizumab**. Cytokines with raw P-value < 0.05 were considered statistically significant. Effect sizes were presented as log2 of fold change where positive (negative) values indicated up-regulation in responders (non-responders). The vertical dashed line represents no difference. The horizontal dashed line represents P-value < 0.05. *Green dot indicates levels of the cytokine are higher in responders; Gray dot ‘None’ indicates levels of that cytokine are not different between responders and nonresponders.*

**
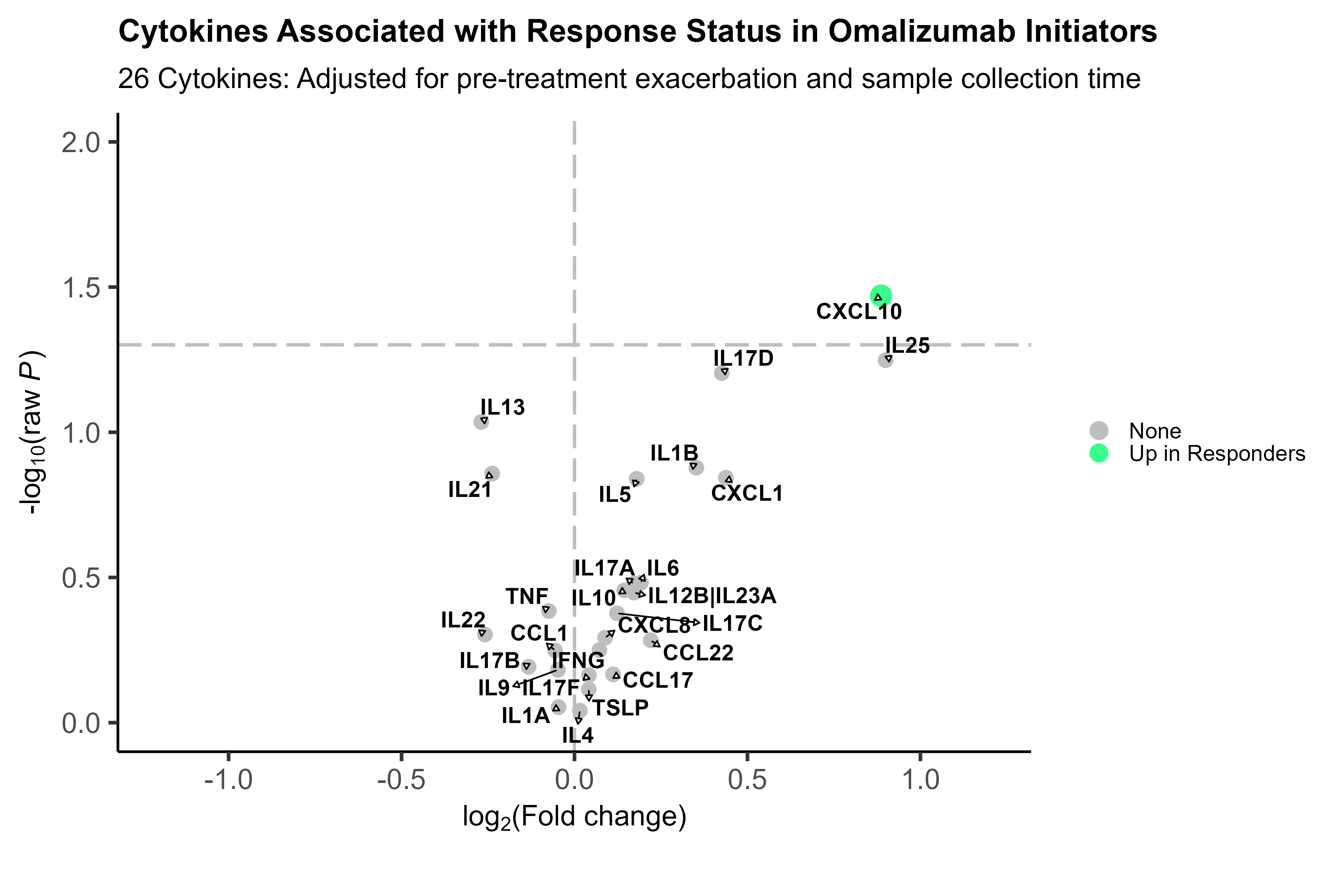
Figure S2. Differential expression analysis for 26 cytokines and response status in the omalizumab group** **adjusted for pre-treatment annualized exacerbation rate and time between sample collection and initiation of omalizumab**. Cytokines with raw P-value < 0.05 were considered statistically significant. Effect sizes were presented as log2 of fold change where positive (negative) values indicated up-regulation in responders (non-responders). The vertical dashed line represents no difference. The horizontal dashed line represents P-value < 0.05. *Green dot indicates levels of the cytokine are higher in responders; Gray dot ‘None’ indicates levels of that cytokine are not different between responders and nonresponders.*

**Figure S3. Top correlation coefficients of pairwise correlations between baseline levels of cytokines or chemokines in mepolizumab responders and non-responders.** The Spearman’s rho was calculated to evaluate the correlation between each pair of cytokines. Blue cells represent positive correlations and red cells negative correlations.

**Figure S4. Top correlation coefficients of pairwise correlations between baseline levels of cytokines or chemokines in omalizumab responders and non-responders.** The Spearman’s rho was calculated to evaluate the correlation between each pair of cytokines. Blue cells represent positive correlations and red cells negative correlations.


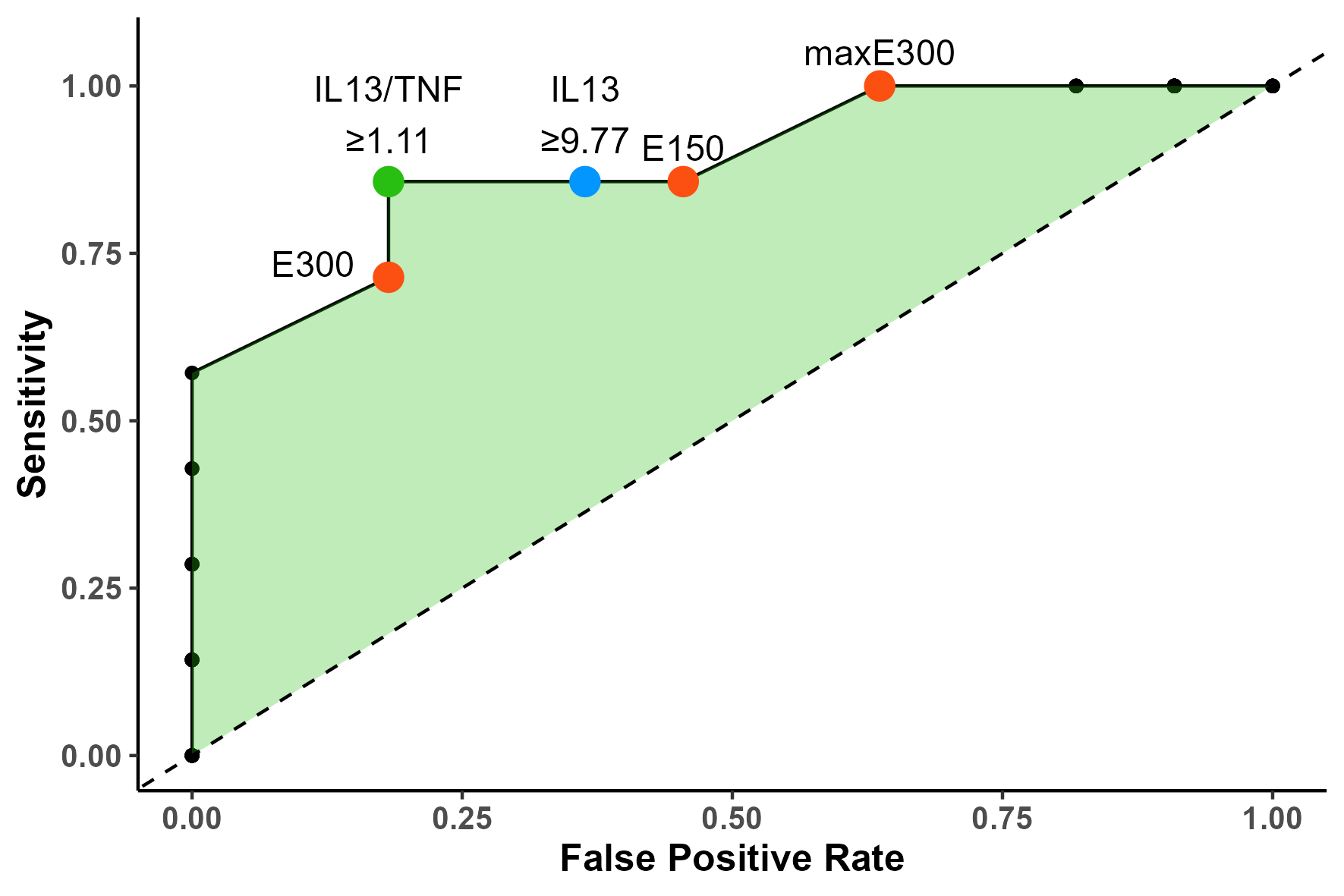


**Figure S5**: The receiver operating characteristic (ROC) curve showing the predictive accuracy (sensitivity and false positive rate (FPR)) of the optimal cut-off points for IL-13 and IL-13/TNF-alpha in differentiating responders from nonresponders to mepolizumab benchmarked against the predictive performance of the baseline eosinophil count of ≥150 (E150) or ≥300 (E300) and the maximum eosinophil counts within one year before mepolizumab initiation of ≥300 (maxE300) cells/μL.

**
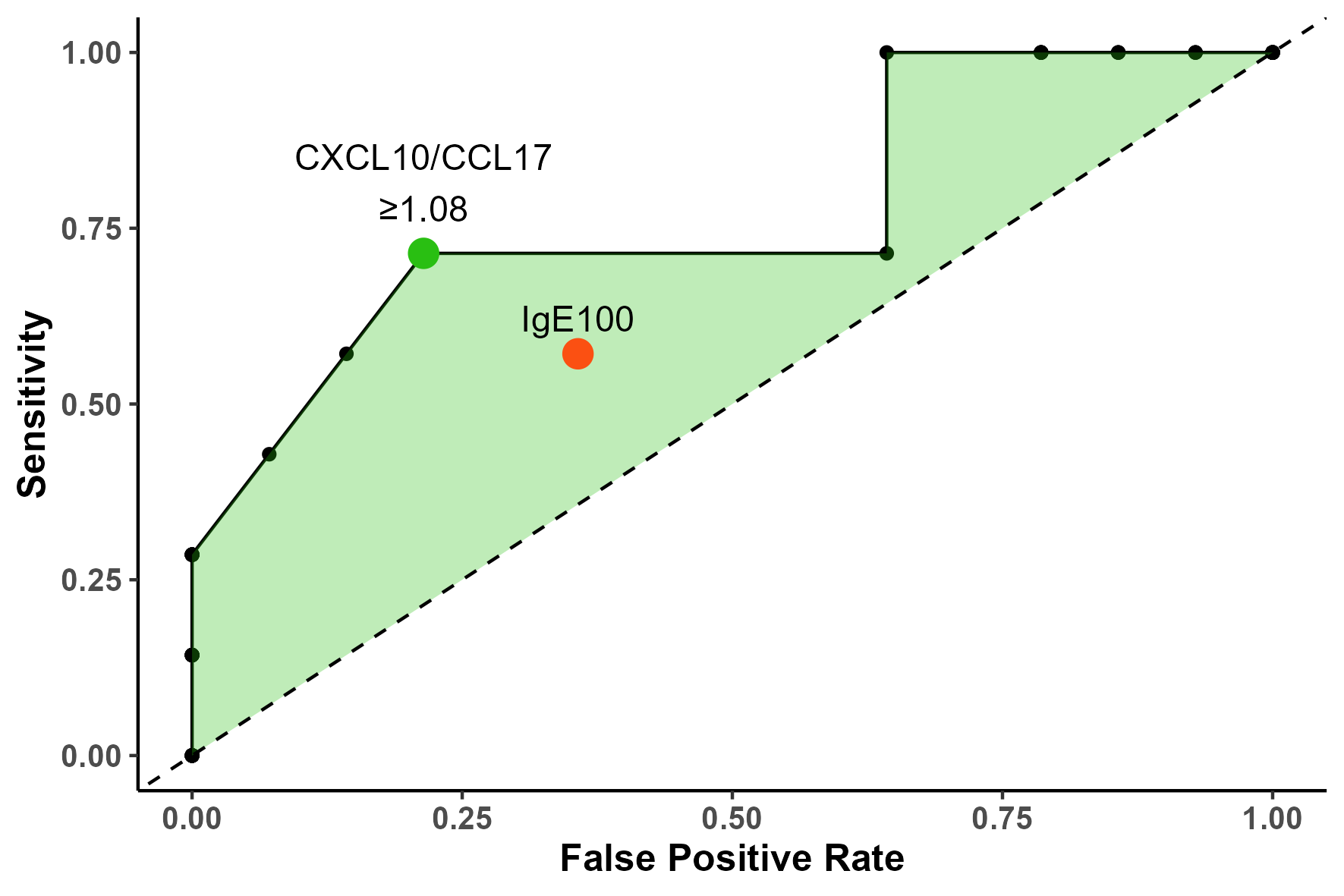
**

**Figure S6**: The receiver operating characteristic (ROC) curve showing the predictive accuracy (sensitivity and false positive rate (FPR)) of the optimal cut-off points for CXCL10/CCL17 in differentiating responders from nonresponders to omalizumab benchmarked against the predictive performance of baseline immunoglobulin E level of ≥100 ku/L (IgE100) cells/μL.
